# Implementing pharmacist-prescriber collaboration to improve evidence-based anticoagulant use: a randomized trial

**DOI:** 10.1186/s13012-023-01273-4

**Published:** 2023-05-15

**Authors:** Shawna N. Smith, Michael Lanham, F. Jacob Seagull, Michael Dorsch, Josh Errickson, Geoffrey D. Barnes

**Affiliations:** 1 Department of Health Management and Policy, School of Public Health, University of Michigan, Ann Arbor, MI USA; 2 Department of Learning Health Sciences, University of Michigan Medical School, Ann Arbor, MI USA; 3 Center for Bioethics and Social Science in Medicine, University of Michigan Medical School, Ann Arbor, MI USA; 4 College of Pharmacy, University of Michigan, Ann Arbor, MI USA; 5 Center for Statistical Consultation and Research, University of Michigan, Ann Arbor, MI USA; 6 Division of Cardiovascular Medicine, Department of Internal Medicine, Institute for Healthcare Policy and Innovation, Frankel Cardiovascular Center, University of Michigan, 2800 Plymouth Rd, Ann Arbor, MI B14 G21448108 USA

## Abstract

**Background:**

Direct oral anticoagulant medications are commonly used to treat or prevent thrombotic conditions, such as pulmonary embolism, deep vein thrombosis, and atrial fibrillation. However, up to 10–15% of patients receiving these medications get unsafe doses based on a patient’s kidney or liver function, potential interactions with other medications, and indication for taking the medication. Alert systems may be beneficial for improving evidence-based prescribing, but can be burdensome and are not currently able to provide monitoring after the initial prescription is written.

**Methods/design:**

This study will improve upon existing alert systems by testing novel medication alerts that encourage collaboration between prescribers (e.g., physicians, nurse practitioners, physician assistants) and expert pharmacists working in anticoagulation clinics. The study will also improve upon the existing alert system by incorporating dynamic long-term monitoring of patient needs and encouraging collaboration between prescribers and expert pharmacists working in anticoagulation clinics. Incorporating state-of-the-art user-centered design principles, prescribing healthcare providers will be randomized to different types of electronic health record medication alerts when a patient has an unsafe anticoagulant prescription. We will identify which alerts are most effective at encouraging evidence-based prescribing and will test moderators to tailor alert delivery to when it is most beneficial. The aims of the project are to (1) determine the effect of notifications targeting existing inappropriate DOAC prescriptions; (2) examine the effect of alerts on newly prescribed inappropriate DOACs; and (3) examine changes in the magnitude of effects over time for both the new prescription alerts and existing prescription notifications for inappropriate DOACs over the 18-month study period.

**Discussion:**

Findings from this project will establish a framework for implementing prescriber-pharmacist collaboration for high-risk medications, including anticoagulants. If effectively implemented at the more than 3000 anticoagulation clinics that exist nationally, hundreds of thousands of patients taking direct oral anticoagulants stand to benefit from safer, evidence-based healthcare.

**Trials registration:**

NCT05351749.

**Supplementary Information:**

The online version contains supplementary material available at 10.1186/s13012-023-01273-4.

Contributions to the literature

• Creating new teams and provider alerts are two commonly cited implementation strategies
• When to use new multi-disciplinary teams and the optimal recipients of provider alerts has not been well studied
• This randomized implementation trial will identify the optimal recipients and timing of alerts for inappropriate medication prescribing
• The trial will also identify which combination of implementation strategies is most likely to lead to improved evidence-based medication prescribing
• Findings from this trial will inform anticoagulation stewardship practices aiming to improve safe, Effective, and evidence-based anticoagulation use

## Introduction

Direct oral anticoagulant (DOAC) medication use is rapidly expanding for six million Americans with common thrombotic conditions. Atrial fibrillation (AF) and venous thromboembolism (VTE) are the leading indications for chronic anticoagulation therapy. Since 2010, four DOAC medications (apixaban, dabigatran, edoxaban, and rivaroxaban) have rapidly overtaken warfarin as the first-line therapy for AF and VTE [1–3]. DOAC therapy is critical to prevent life-threatening stroke and thromboembolic complications.

**Fig. 1:**
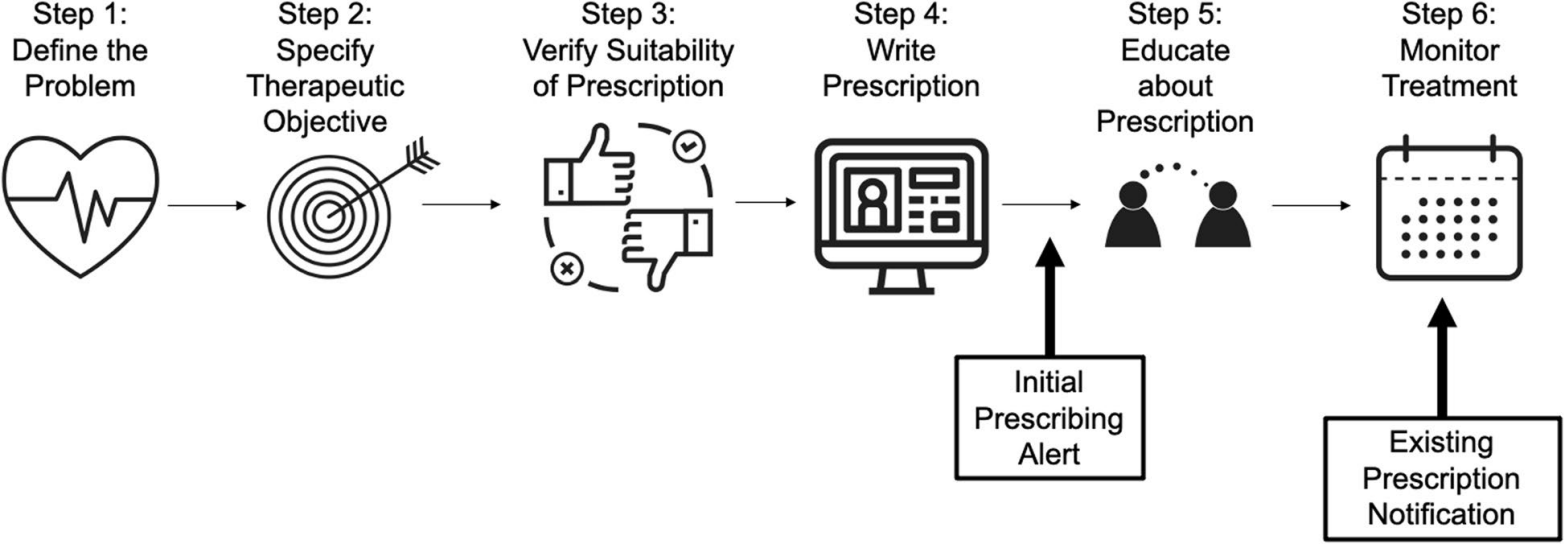
World Health Organization 6 steps of rational prescribing

Despite their popularity, however, unsafe DOAC prescribing is common and dangerous. While initially heralded as easier to prescribe than warfarin, in real-world settings 10–20% of DOAC prescriptions do not follow the Federal Drug Administration (FDA) evidence-based package label instructions [4–6]. The most common reasons for unsafe DOAC use include inappropriate dosing based on renal or liver function, overlooked drug-drug interactions, and dosing based on the wrong indication (e.g., using AF dosing for VTE). These prescribing errors occur both at the time of the initial prescription and in the months to years of follow up (Fig. 1). Data from both large national registries [4, 5] and our local studies[7] confirm a higher rate of bleeding, hospitalization, and death for patients with unsafe DOAC use as compared to evidence-based prescribing. Despite these common prescribing errors, most health systems only provide alerts for potential drug-drug interactions, not for other (more common) causes of inappropriate DOAC prescribing.

While the evidence for how to safely prescribe DOAC medications is well-established, key barriers prevent consistent implementation. Categorized using the Consolidated Framework for Implementation Research (CFIR) [8] taxonomy, these barriers include prescriber knowledge and beliefs (characteristics of individual clinicians), adaptability of evidence-based prescribing to complex clinical cases (intervention characteristics), limited available resources in clinics (inner setting), and challenges with networks and communications in health systems (inner setting). Specifically, many prescribing clinicians are not familiar with the nuances of DOAC dosing or how to apply individual FDA guidance statements for complex patients with multiple issues that may affect dosing (e.g., renal dysfunction and drug-drug interactions) [9, 10]. The prescribing clinicians rarely have ready access to expert pharmacists in their primary care or specialty clinic and anticoagulation clinics cannot manually review every DOAC prescribed across large health systems [11, 12]. Finally, communication between clinical specialists across departments and divisions is always challenging [13].

Theory-based implementation strategies may help to overcome these barriers to evidence-based DOAC prescribing. While more than 70 different implementation strategies have been identified by the Expert Recommendations for Implementing Change (ERIC) project [14], to-date few have been prospectively tested, particularly as they relate to provider-targeted alerts. To address the key barriers to evidence-based DOAC prescribing described above, our study tests two implementation strategies designed specifically to improve evidence-based DOAC prescribing: (1) electronic health record (EHR) medication alerts (combining “remind clinicians” and “develop and implement tools for quality monitoring” strategies from the ERIC taxonomy) and (2) prescriber-pharmacist collaboration (combining “create new clinical teams” and “provide ongoing consultation”).

With respect to EHR tools for implementation, medication alerts are among the most commonly used. However, most medication alerts do not follow key design principles and as such fail to improve patient care. Studies have shown that well-designed medication alerts in the EHR can reduce adverse drug events by up to 50% [15]. However, these promising results are too often not realized, and poorly implemented medication alerts frequently lead to increased clinician dissatisfaction from alert fatigue and habitual override [15, 16]. Currently available DOAC medication alerts suffer from three fundamental design flaws, which together lead to incorrect DOAC dosing both at the time of the initial prescription and at subsequent moments in time where clinical changes should lead to modification of the DOAC medication or dose. These flaws include (1) alerts intrude or interrupt prescriber workflow with low-yield information without actionable tools, often leading to alert dismissal without action; (2) alerts occur only at the time of prescribing, ignoring changes to the clinical scenario that may occur after the initial prescription is written; and (3) alerts do not promote collaboration between prescribers and pharmacists [17]. As a result, many patients receive unsafe prescriptions that can cause significant harm.

Medical informatics experts have proposed key design principles to address these issues (Table 1) [16, 18], which also link into our second strategy of supporting collaborative work between prescribers and pharmacists. Although opportunities for this time of collaborative care are widespread and growing, collaboration to ensure evidence-based prescribing remains an underutilized resource and strategies to encourage the effective use of prescriber-pharmacist collaboration to increase evidence-based prescribing remain untested. In the case of anticoagulant medications, prescribers have long collaborated with more than 3000 anticoagulation clinics nationwide to manage warfarin. Most of these clinics rely on nurses or pharmacists with anticoagulation expertise to manage complex patients and their high-risk medications safely. A 2017 survey of these clinics found that most anticoagulation clinics offer collaborative DOAC care [19]. In this collaborative mode, the pharmacists review prescriptions for appropriateness and for potential drug-drug interactions and recommend appropriate drug/dose selection. However, despite their high volume, DOAC-treated patients accounted for a small fraction (~ 10%) of the overall anticoagulation clinic volume, indicating prescriber underutilization. One exception is the Veterans Health Affairs system, where pharmacist collaboration is common for DOAC prescribing and rates of unsafe DOAC prescribing are significantly lower than outside the Veterans Health Affairs system [20].

**Table 1:** Medication alert design principles [17, 18]

• Improve signal-to-noise ratio by *incorporating clinical context* into alert logic
• Support *collaborative work*, including pharmacists
• Fit within prescriber workflow and mental model, which includes *non-interruptive alerts*
• Display relevant data on why alert occurred
• Ensure system transparency
• Include *actionable tools* within the alert

This study seeks to develop and test two implementation strategies to improve evidence-based DOAC prescribing using a pragmatic comparative effectiveness implementation trial. Prescribers whose patients are receiving inappropriate DOACs will be randomized to different forms of implementation support, including well-designed medication alerts with or without opportunities for prescriber-pharmacist collaboration.

## Design and methods

### Study overview

This pragmatic implementation trial is designed to test the comparative effectiveness of different types of alerts and notifications within the EHR. More specifically, we propose two different interventions for improving DOAC prescribing. The first intervention, intended to target *new* inappropriate DOAC prescriptions, is an automated EHR *alert* that occurs at the time a DOAC medication is prescribed but some potential error exists (e.g., drug-drug interaction, wrong dose for given renal function). All eligible prescribers will be randomized with equal probability to receive either a detailed alert, or the same detailed alert that also includes a referral link for optional DOAC pharmacist review.

The second intervention, intended to target existing inappropriate DOAC prescriptions, is an EHR *notification*. Prescribers with ownership of an inappropriate existing DOAC prescription (i.e., a prescription identified by our system as inappropriate any time *after* the DOAC medication is prescribed when a new potential issue develops [e.g., worsened renal function that impacts dosing, new drug-drug interactions]) will be randomized with equal probability for the notification to be routed either to the prescriber’s inbox or directly to the anticoagulation pharmacist for follow-up.

### Aims and objectives

*Primary aim:* Our study’s primary aim is to determine the effect of the *notifications* targeting existing inappropriate DOAC prescriptions on the proportion of inappropriate DOAC prescriptions that are changed within 7 days. Our primary aim hypothesis is that notifications that are routed directly to the pharmacist rather than the original prescriber will result in a higher proportion of inappropriate prescriptions changed within 7 days.

### Secondary and exploratory aims:

• Aim 2 will examine the effect of the alerts targeting newly prescribed inappropriate DOACs. In Aim 2a, we will examine the overall proportion of alerts that result in a prescription change within 7 days, without accounting for the type of alert received. In Aim 2b, we will examine whether the type of alert (i.e., alert only or alert + pharmacist referral) resulted in a higher proportion of inappropriate DOAC prescriptions changed within 7 days.
• Aim 3 will examine changes in the magnitude of effects over time for both alerts and notifications. In aim 3a, we will examine the change in the proportion of existing inappropriate DOAC prescriptions that trigger notifications that are changed within 7 days over the 18-month course of the study, both overall and by condition. In aim 3b, we will do the same for new DOAC prescriptions that trigger alerts.
• Exploratory analyses will examine potential moderators for alerts and notification conditions, to understand whether there are certain prescribers or patients that benefit most from alerts or notifications that encourage pharmacist collaboration. We will also examine implementation outcomes including patient reach for all types of alerts and notifications, variation in prescriber engagement with alerts/notifications that encourage pharmacist collaboration, and fidelity of prescription changes. Finally, we will assess the effect of the entire system of notifications and alerts on the prevalence of patients with the health system that are receiving inappropriate DOACs.

### Setting

This pragmatic prospective randomized trial will be conducted within one health care system, Michigan Medicine. Michigan Medicine includes more than 4000 clinicians who provided care in over 2.6 million patient clinic visits in fiscal year 2022. The anticoagulation clinic includes four pharmacists and ten nursing staff members who provide care to more than 3000 warfarin-treated patients. In 2020, the anticoagulation clinic staff began monitoring for appropriate DOAC prescribing using a dashboard built within the EHR. A single-day (December 21, 2020) cross-sectional analysis at Michigan Medicine found 9325 patients had DOAC use documented by 1002 primary care, cardiology, hematology, or surgery prescribers (median 23 patients/prescriber, interquartile range [IQR] 7–47). Of these, 670 (7.2%) patients (among 250 unique prescribers) did not follow evidence-based guidelines, with a median of 2 (IQR 1–5) unsafe DOAC prescriptions per prescriber observed on that single-day snapshot.

### Implementation strategies

We will use the following implementation strategies to encourage evidence-based DOAC prescribing. Each strategy is described below, from the perspective of the prescriber, using the Proctor et al. [21] framework (Table 2).

**Table 2:** Implementation strategies

Strategy	Actor(s)	Action(s)	Target of action	Temporality	Dose	Implementation outcome
EHR medication alerts/notifications	EHR	Display well-designed medication alert	*Initial Rx:* Prescriber *Longitudinal monitoring:* Prescriber or Pharmacist	*New Rx:* immediately *Existing Rx:* daily as necessary	Once for each unsafe DOAC prescription	(1) Patient-level adoption of evidence-based DOAC prescribing (2) Effectiveness
Prescriber-pharmacist collaboration	Prescriber and anticoagulation pharmacist	Pharmacist review and recommendation	Prescriber	Referral opportunity immediate; pharmacist review within 3 days	Once for each unsafe DOAC prescription	(1) Patient-level adoption of evidence-based DOAC prescribing (2) Effectiveness

*EHR* electronic health record, *Rx* prescription, *DOAC* direct oral anticoagulant

### EHR medication alerts/notifications

**Fig. 2:**
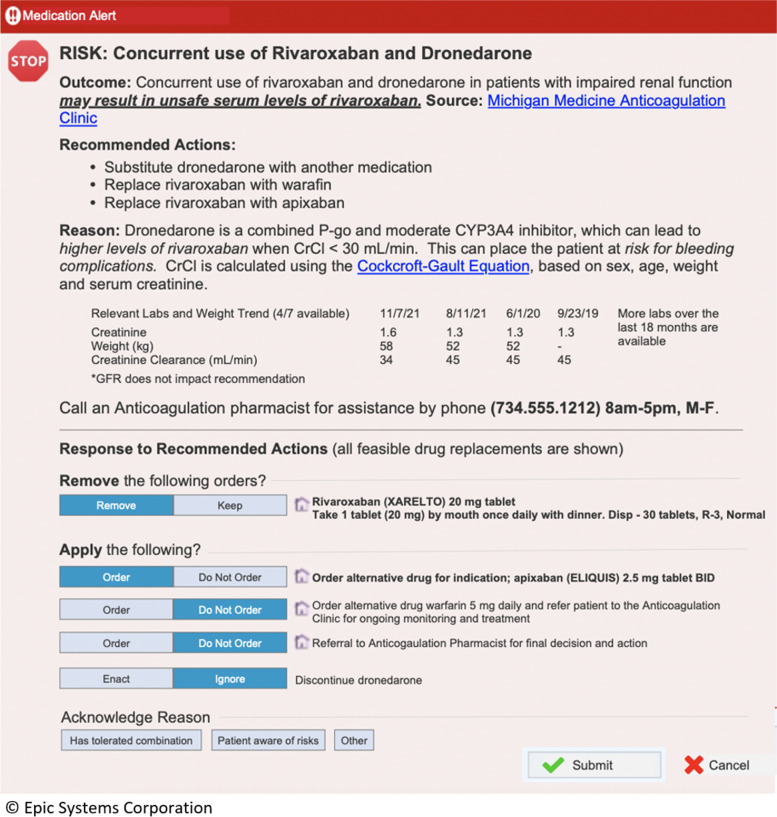
Example dialog box from the alert. Example shows drug-drug interaction between the DOAC rivaroxaban and dronedarone

• For new prescribing errors, prescribers will be shown an EHR alert immediately upon entry of a new prescription that does not meet current evidence-based guidelines. Alerts were designed through a user-centered design process [17] to ensure they are clear and usable. All alerts inform the prescriber of the potential reason for inappropriate prescribing (e.g., drug-drug interaction) and recommended actions the prescriber can take (e.g., ordering an alternative DOAC or another drug) (Fig. 2).
• For existing prescription errors, notification messages alerting personnel to an inappropriate DOAC prescription, as well as clear recommendations for what changes to make to the prescription to align with evidence-based guidelines, will be sent once a day. These notifications were also designed using a user-centered design process (Fig. 2).

#### Prescriber-pharmacist collaboration

Our second strategy for increasing the implementation of evidence-based DOAC prescribing is encouraging prescriber-pharmacist collaboration. This is done in conjunction with the EHR alerts and notifications, described above, but entails different actions. For new prescription alerts, we encourage prescriber-pharmacist by offering a button for prescribers to click within the medication alert that will trigger a request for a DOAC pharmacist to review the prescription (and patient case) and recommend any necessary changes. For existing prescription notifications, prescriber-pharmacist collaboration is encouraged by routing notifications of inappropriate prescriptions initially to DOAC pharmacists directly, rather than prescribers. The pharmacist will then use their expert judgment to determine when it is clinically appropriate to contact the prescriber and request or recommend a prescription change.

### Trial study design

This study is an 18-month prospective randomized clinical trial for alerts and for notifications. Prescribers will be randomized to different types of alerts and/or notifications when a new or existing DOAC prescription is determined to not meet current evidence-based guidelines.

For new inappropriate DOAC prescriptions during the study period: providers who write a new DOAC prescription that does not meet current evidence-based guidelines will be randomized at first instance with equal probability to one of two types of new prescription EHR alerts:

• Alert style 1 will include information about why the prescription is inappropriate as well as recommendations for changing to an evidenced-based prescription.
• Alert style 2 will also include this same information, but will also include a “button” that can be clicked to refer the prescription to a DOAC pharmacist for review.

For existing DOAC prescriptions that are identified as inappropriate, prescribers will be randomized at first instance with equal probability to one of two routings for notifications:

• Notification routing A will route to the prescriber to review the prescription and change it as appropriate.
• Notification routing B will route directly to the DOAC pharmacist for review. The pharmacist may then opt to change the prescription themselves or consult with the prescriber about possible changes.

Note that for our implementation strategies specified above, all four alerts and notifications make use of well-designed EHR alerts; alert style 2 and notification routing B augment this strategy with the encouragement of pharmacist-prescriber collaboration.

Eligible prescribers may be randomized once per condition (alerts, notifications) during the 18-month study duration for alerts and for notifications, immediately following EHR identification of their first inappropriate new (for alerts) or existing (for notifications) DOAC prescription. Prescribers will continue to receive their assigned alert and/or notification type for subsequent inappropriate prescriptions for the duration of the trial. As such, while the trial for alerts and for notifications will be active for the trial in the Michigan Medicine EHR for a total of 18 months, prescribers will not be randomized or receive alerts/notifications until their first inappropriate prescription is identified. A waiver of documented informed consent was approved by the Michigan Medicine Institutional Review Board (IRB). Prescribers were notified prior to the alerts and notifications going live and can opt out at any time during the study period.

### Eligibility and recruitment

#### Prescribers

All Michigan Medicine clinicians with prescribing privileges (including attending physicians, house officers, nurse practitioners, and physician assistants) who see patients in the ambulatory setting and who are not members of the study team will be eligible for study enrollment. Prescribers will be enrolled in the trial and randomized upon having an ambulatory patient whose DOAC prescription triggers an alert (either initial or longitudinal). Prescribers who do not have a new or existing DOAC prescription that is identified as inappropriate during the 18-month trial duration will not be considered eligible for the trial and thus not randomized. We anticipate that 300 prescribers will be enrolled in the trial. All prescribers were notified of the new alert system and companion trial through official EHR communication channels before initiating the trial. Prescribers may opt out of participation at any time.

#### Prescriptions/patients

Our system for assessing DOAC prescription appropriateness will include DOAC prescriptions written in an ambulatory setting for all adult (age ≥ 18) patients who see an eligible prescriber. This includes patients that were initially prescribed a DOAC before the trial commencement but develop unsafe use during the study period. Prescriptions will be excluded if they were written in the Emergency Department or hospital setting (including upon discharge) or in a skilled nursing facility or another institutionalized setting, as prescribers in these settings typically do not follow patients longitudinally and therefore would not be appropriate targets for notifications when prescribing issues develop after the initial prescription is written. Further, inpatient DOAC prescriptions already undergo pharmacy review.

### Comparison to current standard of care

At present, the Michigan Medicine system provides alerts for inappropriate DOAC prescriptions only for new prescriptions and only for select drug-drug interactions (no alerts specific to indication or renal/liver dysfunction). Furthermore, the currently existing alerts do not guide corrective actions. At minimum, our new system will improve upon this by (1) offering enhanced alerts that provide prescribers with additional information on the source of the inappropriate prescription and have been informed by a user-centered design process, and (2) adding existing prescription notifications that ensure that one or more medical professionals are notified when changes in patient characteristics affect the current DOAC prescription appropriateness.

To further ensure patient safety, study investigators have also created a safety review mechanism that will review any un-addressed unsafe DOAC prescribing to ensure that patients are not unnecessarily harmed. This will include a review of any non-evidence-based DOAC prescription at 14 business days following either an alert or notification.

### Randomization

Randomization will occur at the prescriber level and separate, independent randomizations will be performed for new prescription alerts and existing prescription notifications at the time of EHR flagging prescribers' first instance of each. Prescribers may be randomized for alerts, notifications, both, or neither. Once randomized (i.e., after the first alert or notification), alert/notification types for that prescriber will remain consistent throughout the remainder of the study period.

To account for variation in both overall and inappropriate DOAC prescribing occurrence, randomization will be stratified by trainee (vs. non-trainee) status and specialist (e.g., cardiology, hematology) vs. primary care for non-trainees. A permuted block randomization implemented via computer scripts that can interface directly with the EHR will be used to generate stratified random assignments.

This trial is not fully blinded. Prescribers will be aware of the specific content of their assigned alerts and/or notifications but will be unaware of other intervention options. All study staff monitoring outcomes data collection will be blinded to treatment assignment.

### Fidelity monitoring of alerts and notifications

To ensure fidelity of our alert/notification system, a random sample of alerts and notifications will be manually audited quarterly to ensure that they occur when clinically appropriate and contain correct clinical information. Any updates to evidence-based DOAC prescribing guidelines that would affect EHR alert logics (e.g., new drug-drug interactions) will also be continuously monitored, with alert logic updated for all prescribers at least monthly.

### Data safety and monitoring board

The study Data Safety and Monitoring Board (DSMB) will monitor for appropriate clinical management decisions made by prescribers and pharmacists every 6 months during the study period. The DSMB will not report the results of the individual analyses to the study team, but rather will make one of the following recommendations based on their analysis of the data: (1) continue the study without any intervention, (2) provide re-education efforts to both prescribers and pharmacists, or (3) terminate the study due to a concern for patient harm.

### Data

All data used to evaluate alerts and notifications will be pulled from the Michigan Medicine EHR through custom-built reports supplemented by automated and manual chart review. Data will be collected over the 18-month study timeline for alerts and for notifications. Only the trial PIs (Smith, Barnes) and data analysts will have access to the final trial dataset.

### Outcomes and measures

EHR data collection will collect metrics for measuring key outcomes that align with the reach, effectiveness, adoption, implementation, and maintenance (RE-AIM) implementation framework [22], including the adoption of DOAC prescription changes by prescribers following receipt of an alert or notification (primary outcome), reach, clinical effectiveness, prescription change fidelity, and maintenance, as well as some key exploratory outcomes (e.g., pharmacist workload).

### Primary outcome (DOAC prescription changes)

Our *primary outcome* measure is the number (and proportion) of existing medication *notifications* that result in any prescription change within 7 days. Given that clinical complexity prevents every patient from having a clearly defined “correct” DOAC prescription, our primary outcome will assess for any change that is made to the prescription (e.g., dosage, frequency, medication) following delivery of the notification. The 7-day interval was selected to allow time for appropriate anticoagulation clinic pharmacist referral, review, and recommendation to occur.

### Secondary outcomes

#### Prescription changes for new medication alerts

The number (and proportion) of new medication *alerts* that results in any prescription change within 7 days. As with the primary outcome, we will assess for any change that is made to the prescription following delivery of the alert.

Clinical *effectiveness* of our alerts and notifications will be measured by 30-day rates of clinical adverse events, including major [23] and clinically-relevant non-major bleeding (CRNMB) [24] events, as defined by the International Society on Thrombosis and Haemostasis; new or recurrent VTE events; and stroke or systemic arterial embolic events. Adverse events will be captured using two Michigan Medicine-developed health informatics tools, DataDirect and the Electronic Medical Records Search Engine (EMERSE) [25], and independently adjudicated by two expert clinicians (with a third expert available when different opinions arise). DataDirect and EMERSE capture clinical data (e.g., notes, labs, imaging, procedure reports) and allow for rapid identification of populations based on granular clinical details (e.g., demographics, diagnosis, medication use) and/or via text-based searches of the medical record for key terms (e.g., “bleeding”, “stroke”) across pre-defined patient populations. The adjudicators will be blinded as to provider randomization. The 30-day time period was selected to minimize potential contamination from the safety review mechanism described above, and we anticipate that rates for adverse events will be low.

### Exploratory outcomes

*Reach* will be measured by assessing the proportion of DOAC patients that trigger alerts/notifications for inappropriate prescriptions and the proportion whose alerts/notifications are corrected.

*Implementation/fidelity* of alerts/notifications will be defined as (1) for prescribers, how often they order the medication recommended by the alerts/notifications or not (and, for the latter, whether they provide a reason); (2) for pharmacists, how often they respond to referrals from alerts/notifications; and (3) for both prescribers and pharmacists, time from referral/notification to change or recommendation in EHR. Fidelity metrics will be collected via automated chart abstraction using EMERSE [25] and validated by manual chart review of a random selection of 20% of the alerts/notifications that result in prescription changes.

To measure maintenance and sustainment, we will assess over-time changes in reach and adoption outcomes over the full 18-month duration of the study for alerts and for notifications.

### Pharmacist workload

To estimate the pharmacist effort required to manage alerts and notifications, we will calculate the total number of referrals to the anticoagulation clinic during the study period. This will be estimated as the number of referrals/alerts per 1000 DOAC prescriptions. We will then also use tools built within the EHR to measure the time (in minutes) from which a pharmacist views a new alert/notification until one of three actions occur: (1) change in medication prescription is made, (2) alert is dismissed (and reason documented), or (3) message is sent to prescribing clinician. Together, this will allow us to estimate the total pharmacist effort required to manage DOAC alerts/notifications through this system (calculated as full-time equivalents [FTE]) required for a given population size of DOAC-treated patients. To further validate these estimates, DOAC pharmacists will also be asked to document their time spent interacting with and acting upon alerts and notifications during two separate two-week periods using a time-tracking worksheet developed for this study.

### Analyses

#### Primary aim

The primary aim analyses will compare the main effects of the two types of longitudinal notification routings (A vs. B) on our primary outcome, the proportion of patients who have their DOAC prescription changed within 7 days. Mixed-effects logistic regression models will be used to model the probability of changed prescription, with fixed effects for notification type (A vs. B) and stratification variables (resident vs. non, primary care vs. specialist), and any patient-level characteristics that are unbalanced. A prescriber-level random effect will account for patient clustering within prescribers, and an unstructured covariance matrix will be used for residual errors. While we anticipate that few patients will appear in the data multiple times over the 18-month trial period, a patient-level random effect will also be considered, as appropriate.

#### Secondary aims

Aim 2 will examine the effects of the initial alerts for new inappropriate DOAC prescriptions. In Aim 2a, we will examine the proportion of new inappropriate DOAC prescriptions that were changed within 7 days, without distinguishing between alert types. Then, in aim 2b, using a similar modeling approach to that used in the primary aim, we will assess whether patients whose prescribers were randomized to alerts that included DOAC pharmacist referrals were more likely to have their prescription changed than those that received the simple alert (alert 1 vs. 2).

Aim 3 will assess the maintenance of our treatment effects by examining longitudinal change in the effect size for both notifications and alerts. For these analyses, we will extend our initial two-level multi-level model (patients nested in prescribers) used in aims 1 and 2 to a three-level multi-level model that also accounts for time since alerts/notifications were activated. These models will thus include all parameters in aim 1/2, a fixed effect for time in months (0 to 18) since the alerts or notifications were turned on, and an interaction between time and treatment.

### Exploratory analyses

#### Moderators

As DOAC pharmacist time is a limited resource in health care systems, moderator analyses will be used to explore the prescriber- and/or patient-characteristics that most benefit from notifications and alerts that facilitate prescriber-pharmacist collaboration. We hypothesize that both alerts and notifications that engage pharmacists will be more effective at improving change in DOACs prescriptions when:

• Prescribers (1) are based in primary care vs. medical specialists and/or (2) have prescribed fewer DOACs in the six months preceding randomization; and
• Patients (1) are aged 70+; (2) have a VTE (as opposed to AF) diagnosis, as the former has more complex dosing with which many clinicians have less familiarity; (3) have 5+ concurrent medication prescriptions; and/or (4) have moderate or worse renal function (creatinine clearance ≤ 60 ml/min).

Moderators will be assessed by adding interaction effects between indicators for alerts/notifications and the moderator(s) of interest to the analysis models described in aims 1 and 2 above. All moderators will be examined individually initially and all tested moderators will be reported. Those that show both clinical and statistical significance will be considered for use in tailoring alert delivery. Exploratory analyses will be reported using 95% confidence intervals.

#### Implementation outcomes

Descriptive statistics will be analyzed for all pre-defined implementation outcomes, including reach, clinical effectiveness, system-level adoption, implementation, and maintenance. Exploratory bivariate analyses will also look for key sources of variation as they relate to the prescriber or patient characteristics, especially those identified as potential moderators. We will also repeat aims 1 and 2 analyses with clinical effectiveness (including adverse events) and implementation fidelity outcomes to assess whether there are any statistically or clinically significant differences across alert or notification types for any outcomes.

Additional details about additional exploratory analyses, the handling of missing data, sensitivity analyses are included in the Supplemental Appendix.

### Power and sample size

This study is powered for our primary comparison, the comparative effectiveness of two types of longitudinal notification routings (A vs. B) on changes in patient prescriptions. We anticipate 300 prescribers will be randomized (150 per notification), and that prescribers will average two patients triggering a notification. Thus, assuming a patient *n* = 600, prescriber-level intraclass correlation (ICC) of 0.1, and α  = 0.05, we will have 94% power to detect a difference in the proportion of prescriptions changed of 0.40 vs. 0.55 (risk ratio of 1.45) for notification A vs. B. Power for aim 2 analyses of initial alerts will be the same. Power calculations for other outcomes are included in the Supplemental Appendix.

### Trial status

This study was approved by the University of Michigan IRB on March 16, 2022. Initial alerts (and concomitant randomization procedures) for AF went live on August 1, 2022 in a pilot period. Initial alerts and randomization for VTE and longitudinal notifications for both AF and VTE are planned to go live in March 2023, at which point our 18-month trial period will begin for all alerts and notifications.

## Discussion

This pragmatic, EHR-based trial aims to optimize two types of communications related to improving evidence-based DOAC prescribing, with a specific focus on whether, how, and when to deploy communications that encourage collaboration with specialty anticoagulation pharmacists. To our knowledge, this is the first implementation trial related to optimizing safe DOAC prescribing, despite there being more than 3000 anticoagulation clinics in existence across the USA and millions of patients prescribed DOAC medications. Notifications that encourage prescriber-pharmacist collaboration will allow expert pharmacists to review unsafe DOAC use and recommend safe, evidence-based DOAC prescribing. Combining this with a system of novel, well-designed medication alerts has the potential to address multiple barriers to evidence-based DOAC use and receipt, and improve patient clinical outcomes. Exploratory moderators’ analyses will also allow the identification of characteristics of the patients and/or prescribers that most benefit from prescriber-pharmacist collaboration, which will help inform tailored deployment of alerts and notifications encouraging prescriber-pharmacist collaboration, especially in resource-constrained health systems.

This trial is designed to experimentally test two novel implementation strategies to ensure evidence-based DOAC prescribing: (1) EHR-facilitated collaboration between prescribers and pharmacists, and (2) EHR medication alerts/notifications with evidence-based DOAC prescribing information. Both strategies target different implementation barriers that prevent optimal prescriber and health system functioning, but neither strategy has been thoroughly investigated, despite being recommended by health informatics experts [16, 18]. In testing these strategies within a large healthcare system, our study will also add to the literature of randomized studies testing system-wide EHR alerts that have measured robust, multi-faceted implementation outcomes [26–29].

Beyond testing these strategies, however, this study will also inform a broader understanding of the role EHR alerts and health care system communications can play in improving adherence to evidence-based prescribing. A key innovation of this study is the deployment of a system that monitors DOAC patients *longitudinally* and alerts prescribers or pharmacists if unsafe issues develop in the months to years after the initial prescription. Typically, EHR medication alerts focus on initial prescribing errors and are designed to act at one time only. Such alert systems are not designed to assess risks that emerge over time as new diagnoses, medications, laboratory values, and/or treatments accumulate and complicate the clinical scenario. However, our preliminary data indicated that up to 50% of all unsafe DOAC use developed *after* the initial prescription has been written, which led us to design systems and strategies capable of longer-term monitoring. While our trial focuses on testing the comparative effectiveness of different types of notifications, this study will also allow us to better understand how well these longitudinal notification systems can work in practice, and what barriers or unintended consequences to their use might be. We will also be able to evaluate the value of expanding DOAC alerts beyond drug-drug interactions on patient receipt of evidence-based DOACs. Michigan Medicine, like many health care systems, currently has EHR alerts for drug-drug interactions in place; our study will expand alerts to also target inappropriate DOAC dosing based on renal or liver function and inappropriate indications. These new alerts represent the three most common causes of unsafe DOAC prescribing. Again, while our trial analyses will focus on comparing the effects of different EHR alerts, our supplemental, observational interrupted time-series analyses will also allow us to examine the effect of this alert expansion on appropriate DOAC prescribing systemwide.

Our study also has some important limitations. First, given that our study is being conducted within one health care system, results from our analyses may not generalize. However, more than 3000 anticoagulation clinics exist nationwide that can likely benefit from and build on the knowledge generated here. Second, data collection will primarily rely on our EHR, which has known limitations as data are collected for clinical and administrative (rather than research) purposes. This includes concerns about the accuracy and completeness of data and documentation related to DOAC prescribing. We have attempted to minimize this limitation through the manual abstraction processes described above, but recognize that limitations likely still exist. Third, our alerts are limited only to patients prescribed apixaban or rivaroxaban. While these are overwhelmingly the most common DOACs used in the USA, other DOACs (dabigatran and edoxaban) are not being tested explicitly. Finally, it is important to note that because our trial only randomizes prescribers that have a DOAC prescribing error, the causal effects for the alerts and notifications are only relevant conditional on the presence of an error. Thus we are not able to evaluate experimentally whether either alert decreases the overall probability of a future DOAC prescribing error (though we will address this question through our observational ITSA analyses).

## Conclusions

Developing and testing system-wide implementation strategies to improve evidence-based prescribing remains an area ripe for further development in implementation science. DOACs, which have seen rapid recent growth as first-line treatment for AF and VTE, are commonly prescribed inappropriately and present an opportunity for exploring the potential of EHR-based communications to improve evidence-based prescribing, including through the use of DOAC pharmacist collaboration. Findings from this study can inform EHR alert and notification design for other health care systems, improve our understanding of whether and when to engage specialty pharmacists in care, especially for high-risk medications, and improve the long-term health of the patients that are prescribed DOACs.

## Supplementary Information

**Additional file 1.**

## Data Availability

Not applicable.

## Abbreviations

AF: Atrial fibrillation
CFIR: Consolidated Framework for Implementation Research
CRNMB: Clinically relevant non-major bleeding
DOAC: Direct oral anticoagulant
DSMB: Data safety and monitoring board
EHR: Electronic health record
EMERSE: Electronic Medical Records Search Engine
ERIC: Expert Recommendations for Implementing Change
FDA: Food and Drug Administration
FTE: Full-time equivalents
ICC: Intraclass correlation
IQR: Interquartile range
IRB: Institutional review board
ITSA: Interrupted time series analysis
RE-AIM: Reach, effectiveness, adoption, implementation, and maintenance
VTE: Venous thromboembolism
