# Supplementary material for: Implementing pharmacist-prescriber collaboration to improve evidence-based anticoagulant use: a randomized trial
Source: Implement Sci. 2023 May 15;18:16. doi: 10.1186/s13012-023-01273-4 (PMC10184412; doi:10.1186/s13012-023-01273-4)
Supplement: Supplementary file 1 — Additional file 1. [file 13012_2023_1273_MOESM1_ESM.docx]

**Supplemental Appendix**

**Additional Exploratory Analysis Plans**

Carryover effects: We will also assess the potential for differential carryover effects of initial alerts. While Aim 2 analyses will assess whether these alerts differ in whether the patient triggering the alert has their prescription changed, it is also possible that initial alerts that include the option for pharmacist referral vs. those that don’t also differentially affect how many *future* DOAC prescribing errors are made. Specifically, prescriber-pharmacist collaboration may provide the prescriber with new knowledge about evidence-based DOAC prescribing that could be applied to future DOAC prescriptions, avoiding the occurrence of an alert altogether; or, alternatively, prescribers may internalize new information less if they know there is an option to “outsource” the decisions to the pharmacists. To explore these questions, we will also evaluate the main effect of the two initial prescribing alerts on the proportion of DOAC patients that receive a new prescription for an evidence-based DOAC using the model described above, but including all patients to whom they prescribed a DOAC from the point they received their first initial prescription alert through the end of the 18-month study period. This will allow us to evaluate whether correct DOAC prescribing improved overall by type of initial alert.

Evaluation of effectiveness of overall alert system: While our randomized comparisons are designed to determine the best alerts and notifications for improving evidence-based DOAC prescribing through EHR alerts via comparative effectiveness analyses, observational analyses can also evaluate the impact of the introduction of the DOAC EHR alert system *overall* on patient receipt of evidence-based DOACs, relative to before the trial. Using EHR data, we will compute the proportion of patients that were receiving an inappropriate DOAC for each quarter, starting 24 months before the first randomization and continuing 18 months after randomization. Interrupted time series analyses (ITSA) will then be used to compute the change in inappropriate DOAC prescriptions, pre- and post-system introduction, to determine whether the system implemented via the trial resulted in a significant decline in patients on inappropriate DOACs. We will also assess for system-level changes in rates of clinical effectiveness/adverse events. As a further check on changes in DOAC prescribing behaviors in response to the alerts and/or increased prescriber-pharmacist collaboration, we will also use ITSA models to assess for changes in the rate of initial prescribing alerts (per total number of DOACs prescribed) over the 18-month study period, relative to the 24-month period before study start.

Cost analyses: We will perform cost analyses to estimate the cost of anticoagulation pharmacist collaboration. Overall staff costs will be estimated using an average salary and benefits (provided by anticoagulation managers) for the team of anticoagulation clinic pharmacists multiplied by the self-reported time spent managing referrals and alerts (e.g., number of weekly hours dedicated to study activities) divided by 40 work hours/week. Pharmacists will be asked to track the number of hours spent managing referrals and alerts daily using standardized forms. This will be assessed in comparison to the number of alerts/referrals managed by the pharmacists to allow for an estimate of resource requirements for different sized health systems.

***Missing Data and Sensitivity Analyses***

Patients will contribute primary outcome data through EHRs if they stay with Michigan Medicine. As few patients are likely to exit the healthcare system within the short timeframe for primary outcome collection (7 days after alert/notification), missing data should be minimal. Further, patient attrition due to leaving Michigan Medicine also discontinues the ability for their Michigan Medicine prescribers or pharmacists to affect their medication prescription, so does not induce missing data under intent-to-treat principles. Similarly, data on prescribers (e.g., secondary outcome/process data) will continue to be captured via EHR while they are employed at Michigan Medicine. Prescribers that leave Michigan Medicine during the study will be lost to follow-up; however, the Michigan Medicine prescriber population (attending physicians, nurse practitioners, and physician assistants) is generally quite stable and, given the nature of the intervention, we expect attrition to be entirely unrelated to treatment assignment. House officers, while less stable in longevity, do have predictable start and stop dates for patient care, such that missingness is predictable and not related to treatment. Proposed mixed-effects analyses produce valid inferences for data that is missing at random, and multiple imputation will be used as appropriate. If multiple imputation is employed, all analyses will be performed with and without imputed data. Further sensitivity analyses will evaluate the robustness of results under informative missingness scenarios, as appropriate.

While unlikely, a prescriber’s patient panels may change directly or indirectly because of their assigned treatment. To account for this, sensitivity analyses will also rerun models including only patients that were affiliated with the prescriber before randomization for alerts and/or notifications.

***Additional Power and Sample Size Calculations***

For our secondary prescriber-level implementation outcomes (N=300), we will have 94% power to detect a difference in the proportion of prescriptions changed from 0.4 to 0.6 (risk ratio: 1.5). For our patient-level clinical effectiveness secondary outcomes (n=600), we will have 63% power to detect a difference of 1% vs. 0.5% in bleeding rates for both alerts and notifications, assuming N=300 prescribers with an average of 2 patients each, and assuming a prescriber-level intraclass correlation ICC of 0.03.
